# A Case Report Supporting the Use of Teclistamab in Multiple Myeloma With CNS Involvement

**DOI:** 10.1155/crh/8425047

**Published:** 2025-10-28

**Authors:** Jessica Blackman, Justin T. Blackman, Anvita Pauranik, Ashley T. Freeman

**Affiliations:** ^1^Department of Malignant Hematology, British Columbia Cancer Agency, Victoria, British Columbia, Canada; ^2^Faculty of Medicine, University of British Columbia, Vancouver, British Columbia, Canada; ^3^Department of Medical Imaging, Vancouver Island Health Authority, Victoria, British Columbia, Canada; ^4^Department of Medical Imaging, British Columbia Children's Hospital, Vancouver, British Columbia, Canada

**Keywords:** bispecific antibody, case report, central nervous system, multiple myeloma, teclistamab

## Abstract

Central nervous system involvement in multiple myeloma (MM-CNS) is a condition with poor prognosis and no clear treatment options. Standard regimens, including proteasome inhibitors (PIs) and immunomodulatory (IMiD) agents, provide minimal benefit in this setting, highlighting the need for novel therapies. Teclistamab, a bispecific T-cell engager (BiTE) targeting B-cell maturation agent (BCMA) and CD3, has demonstrated robust systemic activity in heavily pretreated MM but its role in CNS disease remains undefined, as patients with CNS involvement have been excluded from pivotal trials. We present the case of a 62-year-old female with high-risk MM who developed extensive leptomeningeal myelomatosis following multiple lines of therapy including autologous transplantation, PI- and IMiD-based regimens, and palliative radiotherapy. Upon presentation with confusion, aphasia, and ataxia, MRI revealed diffuse leptomeningeal enhancement. The patient elected to proceed with teclistamab therapy. Following two cycles, she achieved a very good partial serologic response and MRI demonstrated marked radiologic improvement with resolution of cerebellar nodularity and sulcal enhancement. However, functional recovery was not observed, and the treatment was discontinued after three cycles due to clinical decline and infectious complications. She subsequently transitioned to supportive care and passed away 1 month later. This case report documents one of the first reports of teclistamab demonstrating radiologic improvement in leptomeningeal disease in MM-CNS. While the patient's overall outcome was poor, the observed CNS response supports the biologic plausibility of BiTE penetration and activity in the CNS. These findings suggest the urgent need for prospective studies of BCMA-directed bispecific antibodies in MM-CNS, as well as earlier intervention prior to functional decline.

## 1. Introduction

Central nervous system (CNS) involvement in multiple myeloma (MM-CNS) is an extremely rare complication of MM. It presents either as presumed intracerebral metastases or as leptomeningeal myelomatosis (LMM) [1]. In most instances, it presents with systemic relapse shortly after initial treatment with a very poor prognosis of less than 2 months from the time of diagnosis [2]. Though treatment with proteasome inhibitor (PI)- and immunomodulatory (IMiD)-based regimens may provide some benefit, CNS-MM remains an unmet clinical need particularly for patients who are IMiD and PI refractory [2].

Teclistamab is a recently approved bispecific T cell engager (BiTE) and the first in its class for the treatment of MM. In the nonrandomized Phase I/II MajesTEC-1 trial, teclistamab produced a 63% overall response rate (ORR) in patients who progressed after a standard PI, an IMiD agent, and an anti-CD38 monoclonal antibody at a median follow up of 14.1 months, with 65 patients (39.4%) having a complete response or better [3]. Of the 165 patients who received teclistamab, 77.6% had triple-class refractory disease (median, 5 previous therapy lines) [4]. The Phase 2 portion of the trial is still underway with an estimated study completion date of June 26, 2026 [5]. However, patients with MM-CNS were excluded from this trial and so these results will not shed light on potential CNS activity of anti-B-cell maturation agent (BCMA) BiTEs.

Here, we report a case of teclistamab therapy inducing a partial response in leptomeningeal disease in a patient with MM-CNS.

## 2. Case Presentation

A 62-year-old woman was diagnosed with MM in the winter of 2020 after presenting with hyperviscosity, multiple bone lesions, pathological fracture of the right femur, acute kidney injury, and anemia. The bone marrow aspirate reported plasma cell myeloma with plasmablastic morphology. The bone marrow biopsy was nondiagnostic. Cytogenetics revealed high-risk changes including 1q amplification and 17p deletion. Light chain/paraprotein levels at diagnosis are not available. She received 5 cycles of cyclophosphamide, bortezomib, and dexamethasone, followed by melphalan-conditioned tandem autologous stem cell transplants 6 months after her initial diagnosis. She went on to receive maintenance therapy with lenalidomide and ixazomib. Best response was a very good partial response (IgG paraprotein 3.0 g/L and normal serum free light chains) achieved during the maintenance period. Ixazomib was stopped after 2 years with lenalidomide maintenance continued until progression. She completed 2 years of monthly zoledronic acid treatments.

Three and a half years following initial MM diagnosis, she presented with sacral pain and neurologic symptoms in the lower extremities. MRI of the lumbar spine showed extramedullary disease at S2 resulting in nerve root impingement and encasement of the sacral nerve roots. The extraosseous soft tissue extension measured 2.5 × 1.6 × 3.4 cm. IgG kappa paraprotein was 6.1 g/L, and the level of kappa light chains was 32 mg/L. She received palliative radiation to the sacral disease followed by 6 cycles of isatuximab, carfilzomib, and dexamethasone. Best serologic response was stable disease with this regimen. Following Cycle 6, she experienced serologic progression (IgG kappa paraprotein of 15.2 g/L and kappa light chains of 114 mg/L) and extramedullary progression with CT showing bilateral soft tissue masses around the femurs. She received further palliative radiation to the bilateral femurs in winter 2024 and was transitioned to pomalidomide, cyclophosphamide, and dexamethasone. In total, she received 7 cycles prior to serologic and extramedullary progression.

**Figure 1:**
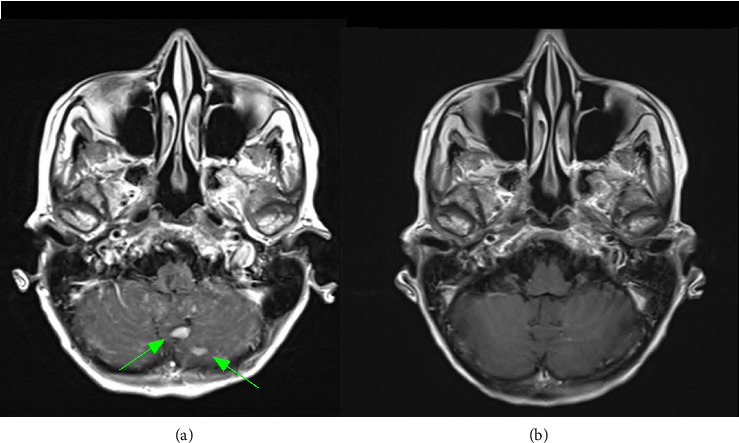
Improvement in nodular leptomeningeal enhancement. Post gadolinium axial T1 sequence images. (a) Before treatment with teclistamab, repetition time (TR): 2000.0 ms, echo time (TE): 8.7 ms, and echo train length (ETL): 5. (b) After treatment with teclistamab, TR: 2000.0 ms, TE: 12.0 ms, and ETL: 5. Initial large nodular enhancements around the vermis (green arrows) show dramatic improvement following treatment.

**Figure 2:**
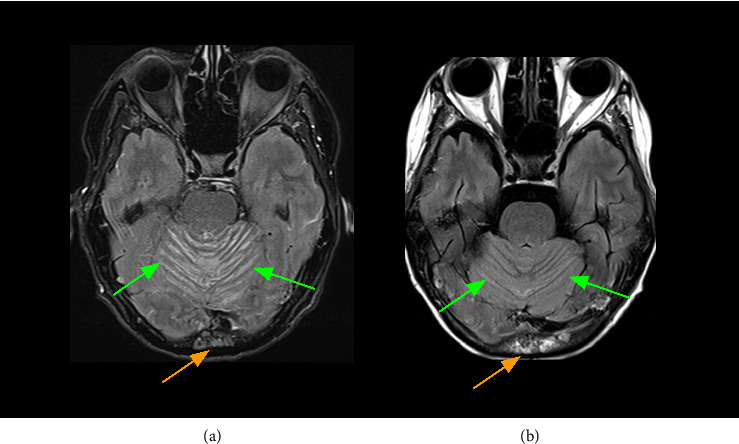
Improvement in cerebellar sulcal enhancement. Post gadolinium axial T2 fluid-attenuated inversion recovery (FLAIR) images. (a) Before treatment with teclistamab, deep resolve boost (DRB): yes, repetition time (TR): 9400.0 ms, echo time (TE): 81.0 ms, and echo train length (ETL): 21. (b) After treatment with teclistamab, DRB: no, TR: 9000.0 ms, TE: 83.0 ms, and ETL: 17. Initial diffuse leptomeningeal enhancement along the cerebellar folia shows dramatic improvement following treatment (green arrows). Orange arrows demonstrate one of the several osseous lesions due to multiple myeloma.

In the fall of 2024, she presented to the Emergency Room with new neurologic symptoms including confusion, aphasia, and ataxia. MRI brain showed diffuse leptomeningeal involvement, most marked in the cerebellum with nodular (Figure 1(a)) and sulcal (Figure 2(a)) enhancement. There was also mildly increased FLAIR signal along the cerebral sulci with no definitive concerning parenchymal enhancement. MRI sacrum showed progressive disease involving the L5 vertebral body and sacrum with extraosseous extension seen involving the right sacral ala. The soft tissue encased the exiting right L5 nerve root.

Best supportive care was the initial recommendation of the oncology team due to poor functional status and limited treatment options. However, clinical status improved with high-dose dexamethasone, and a trial of teclistamab was initiated at the patient's request. ECOG performance status was 3 at the time of teclistamab start. A repeat MRI brain was done to establish a new baseline with the start of teclistamab and demonstrated progression in the leptomeningeal disease from the MRI several weeks prior. Laboratory investigations just prior to teclistamab initiation demonstrated the following: IgG kappa paraprotein 21.5 g/L, kappa light chains 182.3 mg/L, beta-2-microglobulin 3.0 mg/L, albumin 21 g/L and LDH 13 73 U/L (ULN 246 U/L), hemoglobin 99 g/L, platelets 144 × 10^9^/L, absolute neutrophil count 13.55 × 10^9^/L. MRD testing was not completed prior to or following teclistamab as this test is not available at our institution. Cerebrospinal fluid (CSF) studies were not done due to clear CNS involvement on radiological imaging. The patient did not receive any intrathecal therapies, radiotherapy, or systemic agents alongside teclistamab. She completed Cycle 1 with no symptoms of cytokine release syndrome or immune effector cell–associated neurotoxicity syndrome. She received a total of 3 cycles. There were several treatment delays due to recurrent infections despite intravenous immunoglobulin likely due to repeated aspiration events in the setting of severe secondary immune suppression. Best serologic response was a very good partial response (IgG kappa paraprotein 5.5 g/L, kappa light chains 3.7 mg/L). Repeat MRI following Cycle 2 showed substantial improvement of areas of enhancement with resolution of the previously noted cerebellar nodularity (Figure 1(b)) and sulcal enhancement (Figure 2(b)). CSF studies had not yet been planned as we were too soon into treatment. MRI following Cycle 3 did not show further improvement. Unfortunately, the initial radiologic improvement in leptomeningeal disease did not translate into functional recovery and the patient remained hospitalized. The decision was made to transition to best supportive care following Cycle 3, and the patient passed away a month later.

## 3. Discussion

MM-CNS is a rare condition with poor prognosis and no clear standard treatment. Various treatment approaches have been described in case reports including IMiD- and PI-based regimens, systemic chemotherapy with CNS penetration, intrathecal chemotherapy, and radiation [1, 2, 6].

The known blood–brain barrier permeability of current myeloma treatments has been previously summarized [7]. Teclistamab is a bispecific antibody that targets both CD3 expressed on the surface of T cells and BCMA expressed on the surface of myeloma cells, mediating T-cell activation and subsequent lysis of BCMA-expressing myeloma cells. At this time, there is no available information describing its ability to permeate the blood–brain barrier, but there was mild response in patients with extramedullary disease suggesting some penetration [8]. BiTEs are small antibody-derived molecules, ∼55 kDa for classical BiTEs compared to full-sized antibodies at ∼150 kDa. Their smaller size gives them theoretic potential to cross the blood–brain barrier, but this is highly variable and not considered to be generally efficient under normal conditions [9]. T-cell activation and cytokine release may increase blood–brain barrier permeability allowing for CNS access by BiTEs [9].

Indeed, there is clinical evidence for CNS activity of BiTEs. Blinatumomab is a BiTE targeting CD19 and CD3 and is the smallest BiTE at 55 kDa. It has demonstrated some CNS activity in its use to treat B-cell acute lymphoblastic leukemia (B-ALL) with CNS involvement [10]. Glofitamab is a BiTE targeting CD20 and CD3 and has demonstrated activity in both secondary CNS lymphoma and relapsed primary CNS lymphoma [11]. It has been shown to penetrate the blood–brain barrier and facilitate the infiltration of T cells into the CNS tumors through the combination of direct T-cell tumor cell synapse formation, cytokine production, and peripheral T-cell recruitment, leading to clinical response [11].

Elranatamab is a BiTE very similar to teclistamab targeting BCMA-expressing MM cells and CD3-expressing T cells. Its ability to yield a complete remission was documented in the case of a 37-year-old male patient with MM-CNS treated with elranatamab as a fourth-line therapy [12]. This patient was reported the longest case of functional complete remission since diagnosis.

A review of the literature on bispecific antibody treatment in patients with MM-CNS revealed a notable paucity of data. Two congress posters presented at the European Hematology Association (EHS) 2024 meeting described clinical outcomes of bispecific antibody treatments in patients with MM-CNS. One evaluated 13 patients with MM-CNS who were treated with either bispecific antibodies (teclistamab and talquetamab) or CAR-T cell therapy (idecabtagene vicleucel) [13]. Five patients received teclistamab and talquetamab for overt CNS relapse but showed no objective responses with a poor median overall survival (OS) of 4.8 months from CNS diagnosis. In contrast, 8 patients were treated with a multimodal consolidation approach consisting of PACE-like chemotherapy, intrathecal therapy, and/or radiotherapy, followed by novel immunotherapies (teclistamab, talquetamab, or idecabtagene vicleucel). This group demonstrated a more favorable CNS relapse-free survival of 13.4 months, suggesting potential benefit from this strategy. A note was made of a patient with meningeal myelomatosis supporting the direct effect of teclistamab on the control of CNS disease where the patient showed a significant increase in CSF T-cells while the plasma cell count decreased post teclistamab and is actively still in asymptomatic remission. The second poster analyzed relapsed/refractory MM-CNS treated with teclistamab or elranatamab [14]. Nine patients were treated (teclistamab: 8; elranatamab: 1), with data for 8. The median patient age was 60 years, with a median disease duration of 3 years and a median of 3 prior treatment lines. Among 6 patients with available cytogenetics, 50% had high-risk MM. CSF analysis in 7 patients was abnormal. Brain MRI carried out in all 8 patients showed 1 with pachymeningitis and 1 with intraparenchymal lesions. Three patients received additional treatment: radiotherapy (1), methotrexate IT (1), and thiopeta IT (1). At least two patients showed both systemic and neurological responses to bispecific therapy, with one patient maintaining a complete remission and neurological improvement after 1 year.

Although our patient achieved a radiologic response in CNS disease following teclistamab, the treatment was stopped due to deteriorating functional status. This was multifactorial due to poor functional status at the start of therapy and infectious complications. Unfortunately, the patient passed away within a month of stopping the treatment. This highlights the importance of initiating complex treatments before functional decline when possible.

## 4. Conclusion

We describe a 62-year-old woman with high-risk multiple myeloma complicated by extensive leptomeningeal disease. Teclistamab therapy resulted in radiologic improvement of leptomeningeal disease, supporting the use of teclistamab for CNS involvement in multiple myeloma.

## Data Availability

The data that support the findings of this study are available on request from the corresponding author. The data are not publicly available due to privacy or ethical restrictions.
